# Clinicopathologic analysis of nodal T-follicular helper cell lymphomas, a multicenter retrospective study from China

**DOI:** 10.3389/fimmu.2024.1371534

**Published:** 2024-03-27

**Authors:** Shanshan Ma, Suxiao Li, Xiaona Zuo, Wencai Li, Lifu Wang, Weiping Liu, Zhe Wang, Wei Sang, Yanjie Wang, Xudong Zhang, Mingzhi Zhang

**Affiliations:** ^1^ Department of Oncology, Lymphoma Diagnosis and Treatment Center of Henan Province, The First Affiliated Hospital of Zhengzhou University, Zhengzhou, China; ^2^ Department of Pathology, Beijing Boren Hospital, Beijing, China; ^3^ Department of Pathology, The First Affiliated Hospital of Zhengzhou University, Zhengzhou, China; ^4^ Department of Pathology, Henan Province People’s Hospital, Zhengzhou, China; ^5^ Department of Pathology, Department of Pathology, West China Hospital of Sichuan University, Chengdu, China; ^6^ Department of Pathology, Department of Pathology, Xijing Hospital, the Fourth Military Medical University, Xi′an, China; ^7^ Department of Hematology, Affiliated Hospital of Xuzhou Medical University, Xuzhou, Jiangsu, China

**Keywords:** nodal T-follicular helper cell lymphoma, peripheral T-cell lymphoma, clinicopathology, clinical features, prognostic analysis

## Abstract

**Background:**

Nodal T-follicular helper cell lymphomas (nTFHLs) represent a new family of peripheral T-cell lymphomas (PTCLs), and comparative studies of their constituents are rare.

**Methods:**

This study retrospectively enrolled 10 patients with nTFHL-F and 30 patients with nTFHL-NOS diagnosed between December 2017 and October 2023 at six large comprehensive tertiary hospitals; 188 patients with nTFHL-AI were diagnosed during the same period at the First Affiliated Hospital of Zhengzhou University for comparison.

**Results:**

Compared with nTFHL-AI, nTFHL-NOS patients exhibited better clinical manifestations, lower TFH expression levels, and a lower Ki-67 index. However, no differences in clinicopathological features were observed between nTFHL-F and nTFHL-AI patients as well as nTFHL-NOS patients. According to the survival analysis, the median OS for patients with nTFHL-NOS, nTFHL-AI, and nTFHL-F were 14.2 months, 10 months, and 5 months, respectively, whereas the median TTP were 14 months, 5 months, and 3 months, respectively. Statistical analysis revealed differences in TTP among the three subtypes(*P*=0.0173). Among the population of patients receiving CHOP-like induction therapy, there were significant differences in the OS and TTP among the nTFHL-NOS, nTFHL-AI, and nTFHL-F patients (*P*=0.0134, *P*=0.0205). Both the GDPT and C-PET regimens significantly improved the ORR, OS, and PFS in nTFHL patients.

**Conclusion:**

There are significant differences in the clinical manifestations, pathology, and survival outcomes among the three subtypes of nTFHLs. However, further research with a larger sample size, and involving clinical pathology and molecular genetics is needed to determine the distinctive biological characteristics of these tumors.

## Introduction

Follicular helper T (TFH) cells are a subset of CD4-positive T cells crucial for germinal center (GC) formation, high-affinity antibody production, and memory B-cell development (1). According to the 2016 revision of the World Health Organization (WHO) classification of lymphoid neoplasms, TFH-originated nodal peripheral T-cell lymphomas (nPTCLs) were classified based on the expression of at least two or three TFH markers, such as CD279/PD1, CD10, BCL-6, CXCL13, and ICOS. These tumors were categorized as angioimmunoblastic T-cell lymphoma (AITL), follicular peripheral T-cell lymphoma (F-PTCL), or nodal peripheral T-cell lymphoma with TFH phenotype (nPTCL-TFH) (2). Studies have shown that AITL, F-PTCL, and nPTCL-TFH express the same TFH-related antigens and exhibit similar molecular abnormalities (3–6). Furthermore, gene mutations commonly associated with AITL, such as RHOA, TET2, and DNMT3A, have also been identified in patients with F-PTCL and nPTCL-TFH (7–9). Currently, the classification of this family is based mainly on histopathological features. According to the 5th edition of the WHO Classification of Hematolymphoid Tumors: Lymphoid Neoplasms, nTFHLs were renamed the nTFHL angioimmunoblastic type (nTFHL-AI), nTFHL follicular type (nTFHL-F), and nTFHL not otherwise specified (nTFHL-NOS) (10). However, because of the rarity of nTFHL-F and nTFHL-NOS, research comparing the clinical and pathological features of the three subtypes of nTFHLs is limited. In this study, we analyzed the pathological and clinical features of the three subtypes of nTFHLs.

## Materials and methods

### Study population

The data from patients with nTFHL-F or nTFHL-NOS were obtained from six large comprehensive tertiary hospitals in China spanning from December 2017 to October 2023 (**Supplementary Table S1** for participating institutions). The clinical data are summarized in **Table 1**. Patients with nTFHL-AI diagnosed at the First Affiliated Hospital of Zhengzhou University during the same period were also included for comparison. The follow-up cutoff date was October 30, 2023. The study was conducted in accordance with the Declaration of Helsinki and approved by the ethics review board.

**Table 1:** Characteristics of nTFHL-F and nTFHL-NOS patients.

Category	Age (years)	Sex	Stage	Extra-node invasion	Firstline treatment and effect	Latest status	TTP (months)	OS (months)
nTFHL-F	70	F	IV	Bone marrow	Treatment not received	Dead	NA	3
nTFHL-F	61	M	IV	Skin, Bone marrow	CHOP, PD	Dead	3	3
nTFHL-F	66	M	IV	Bone marrow	miniCHOP, PD	Dead	0.5	0.5
nTFHL-F	55	F	NA	None	Unknown	Alive	NA	37
nTFHL-F	65	F	IV	Bone marrow	CHOPE, PR	Alive	9	9
nTFHL-F	77	M	III	None	CHOPE	Dead	0.6	0.6
nTFHL-F	58	M	IV	Parotid gland, Skin, Bone marrow	CHOPE, PD	Dead	1.2	5
nTFHL-F	68	F	IV	Muscle, Bone marrow	BV + CHP, Not evaluated	Alive	1.8	1.8
nTFHL-F	52	F	IV	Skin	Treatment not received	Dead	NA	15
nTFHL-F	52	F	III	None	CHOPE, PR	Dead	5.4	6
nTFHL-NOS	43	M	II	Muscle	CHOPE, CR	Alive	19.6	20.1
nTFHL-NOS	59	F	IV	Liver, Bone marrow	CVP, PD	Alive	4.4	24.8
nTFHL-NOS	53	M	III	None	CVP + Aza, CR	Alive	13.1	20.7
nTFHL-NOS	63	F	III	None	GDPT, PR	Alive	28.2	28.2
nTFHL-NOS	73	F	IV	Pharynx, Tongue, Bone marrow	GDPT, PD	Dead	14	14
nTFHL-NOS	53	M	IV	Pharynx, Intestine, Bone marrow	CHOP, PR	Dead	4.4	4.4
nTFHL-NOS	39	F	II	None	CHOP + Tha, PD	Alive	2.8	63.3
nTFHL-NOS	72	M	III	None	GDPT, PR	Dead	6.7	11.5
nTFHL-NOS	74	M	IV	Stomach, Bone marrow	CHOP, PR	Alive	19.2	19.2
nTFHL-NOS	32	M	IV	Pharynx, Skin, Bone marrow	GDPT, PR	Dead	55.5	55.5
nTFHL-NOS	67	M	II	Tongue	Unknown	Dead	13	13
nTFHL-NOS	74	F	I	None	CHOP, CR	Alive	6	6
nTFHL-NOS	11	M	II	None	CHOP, CR	Alive	12.5	12.5
nTFHL-NOS	77	M	III	None	Das + AZA + CEP, PD	Dead	1.1	1.1
nTFHL-NOS	32	M	IV	Bone, Bone marrow	EPOCH, PD	Alive	3.7	13.8
nTFHL-NOS	79	M	IV	Pharynx, Lung, Bone, Skin	Treatment not received	Dead	NA	0.6
nTFHL-NOS	70	M	III	Pharynx	miniCHOP + Chi, PD	Dead	4	4
nTFHL-NOS	65	F	IV	Pharynx	CHOPE, PD	Alive	1.3	9.8
nTFHL-NOS	52	M	III	None	CHOPE, PR	Dead	14.2	14.2
nTFHL-NOS	72	M	IV	Pharynx, Duodenum	BV + CHP, PD	Dead	4	4.4
nTFHL-NOS	65	M	IV	Bone marrow	Treatment not received	Dead	NA	0.2
nTFHL-NOS	50	M	IV	Skin, Bone	CHOP, PR	Dead	12.5	12.5
nTFHL-NOS	68	F	IV	Pharynx, Skin	CHOPE + Chi, PD	Dead	4	7.3
nTFHL-NOS	60	M	I	None	CHOP + AZA, CR	Alive	5.2	5.2
nTFHL-NOS	86	F	Unknown	Unknown	COP, PR	Alive	6	6
nTFHL-NOS	40	M	IV	Adrenal glands	CHOP + Chi, CR	Dead	11.5	11.5
nTFHL-NOS	59	M	III	None	CHOPE, PR	Alive	7.4	6.4
nTFHL-NOS	55	F	IV	Unknown	GDPT, PD	Alive	5.4	12.6
nTFHL-NOS	67	F	IV	Pharynx, Bone marrow	BV + CHP, SD	Alive	13	13
nTFHL-NOS	60	M	II	Pharynx	Das + AZA + CEP, Not evaluated	Alive	0.1	0.2

F, Female; M, Male; CHOP, Cyclophosphamide, Doxorubicin, Vincristine, Prednisone; miniCHOP, Dose-reduced CHOP; CHOPE, CHOP + Etoposide; COP, Cyclophosphamide,

Vincristine, Prednisone; CVP, Cyclophosphamide, Doxorubicin, Vincristine, Prednisone; BV + CHP, Brentuximab vedotin + Cyclophosphamide, Doxorubicin, Prednisone; GDPT, Gemcitabine,

Cisplatin, Prednisone, Thalidomide; EPOCH, Etoposide, Vincristine, Doxorubicin, Cyclophosphamide, Prednisone; Das + AZA + CEP, Dasatinib, Azacitidine, Etoposide, Cyclophosphamide,

Prednisone;Tha, Thalidomide; Chi, Chidamide; NA, not available.

### Histopathology and immunohistochemistry

**Figure 1:**
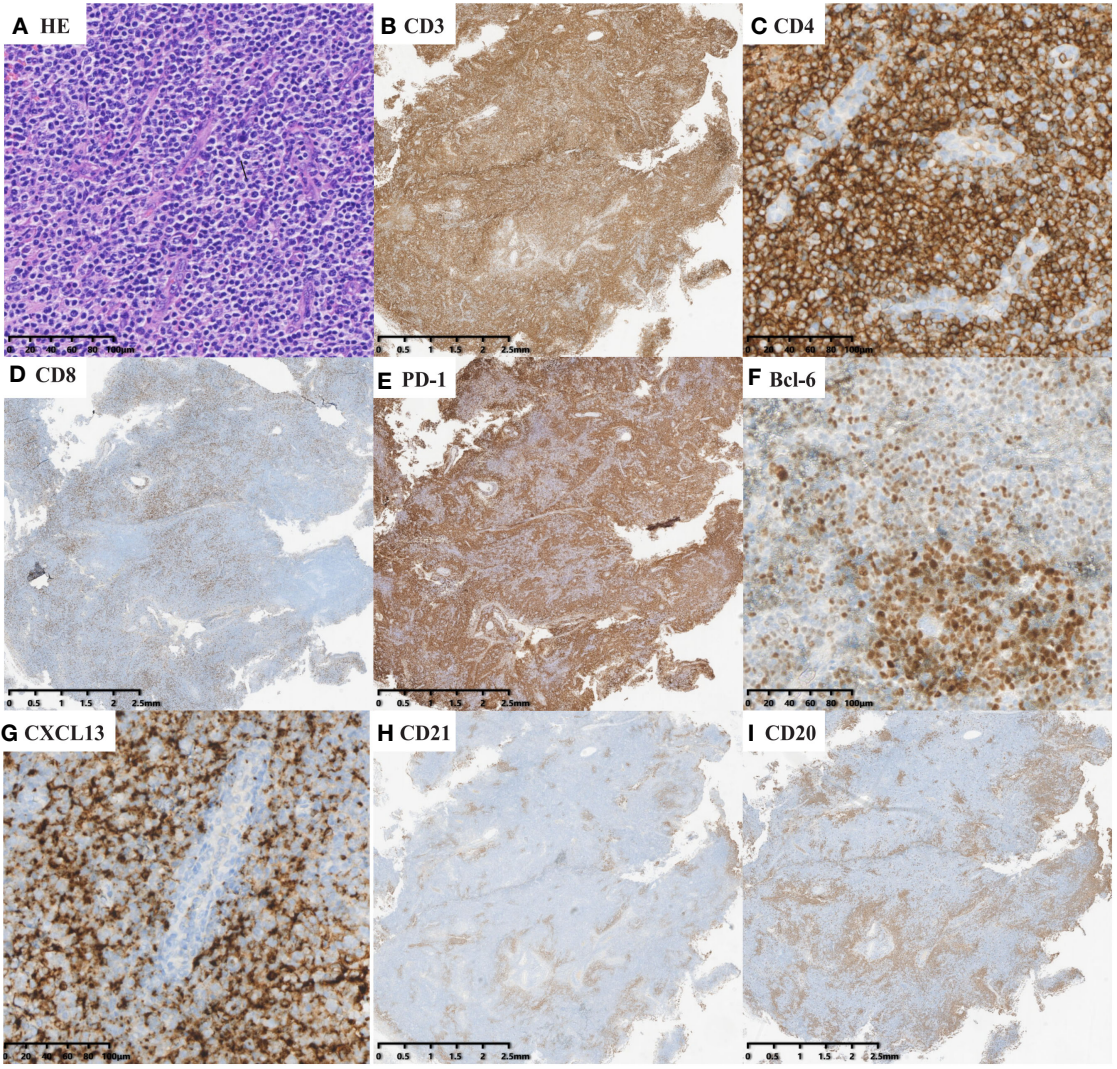
nTFHL-AI: H&E shows that the neoplasm is diffuse and composed of a heterogeneous cell infiltrate associated with numerous HEVs, inflammatory cells (**A** × 200). The tumor cells are positive for CD3 (**B** × 10), CD4 (**C** × 200), PD-1 (**E** × 10), BCL-6 (**F** × 200) and CXCL13 (**G** × 200) and negative for CD8 (**D** × 10). CD21 (**H** × 10) highlights hyperplastic and dilated FDC networks.

**Figure 2:**
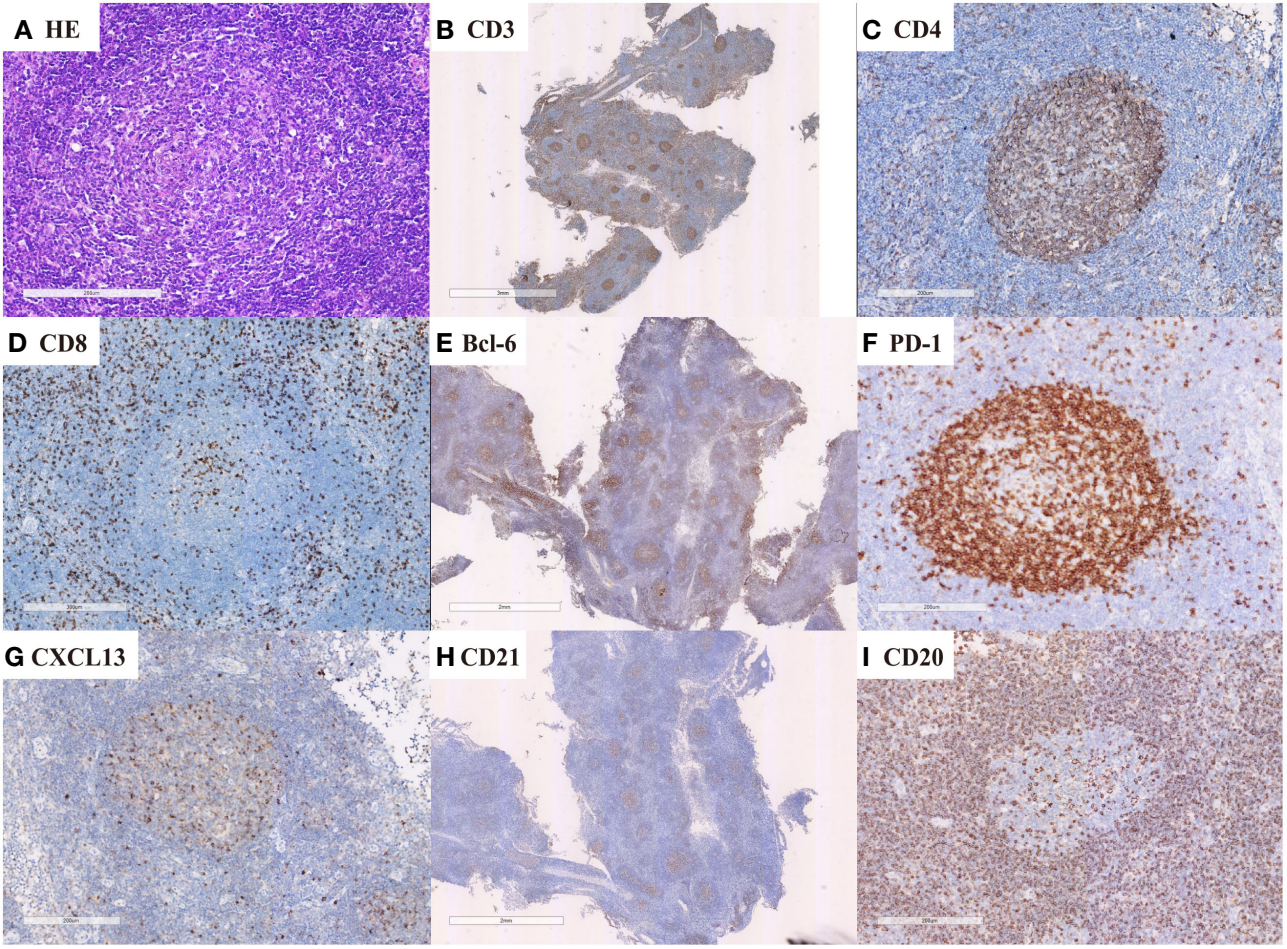
nTFHL-F: H&E shows effacement of lymph nodal architecture by monomorphic nodules (**A** × 100); the nodule comprises small to intermediate size tumor cells. These cells are immunopositive for CD3 (**B** × 10), CD4 (**C** × 100), BCL-6 (**E** × 10), CXCL13 (**G** × 100) and PD-1 (**F** × 100) while negative for CD8 (**D** × 100). CD21 staining shows mild expansion of FDC meshworks (**H** × 10).

**Figure 3:**
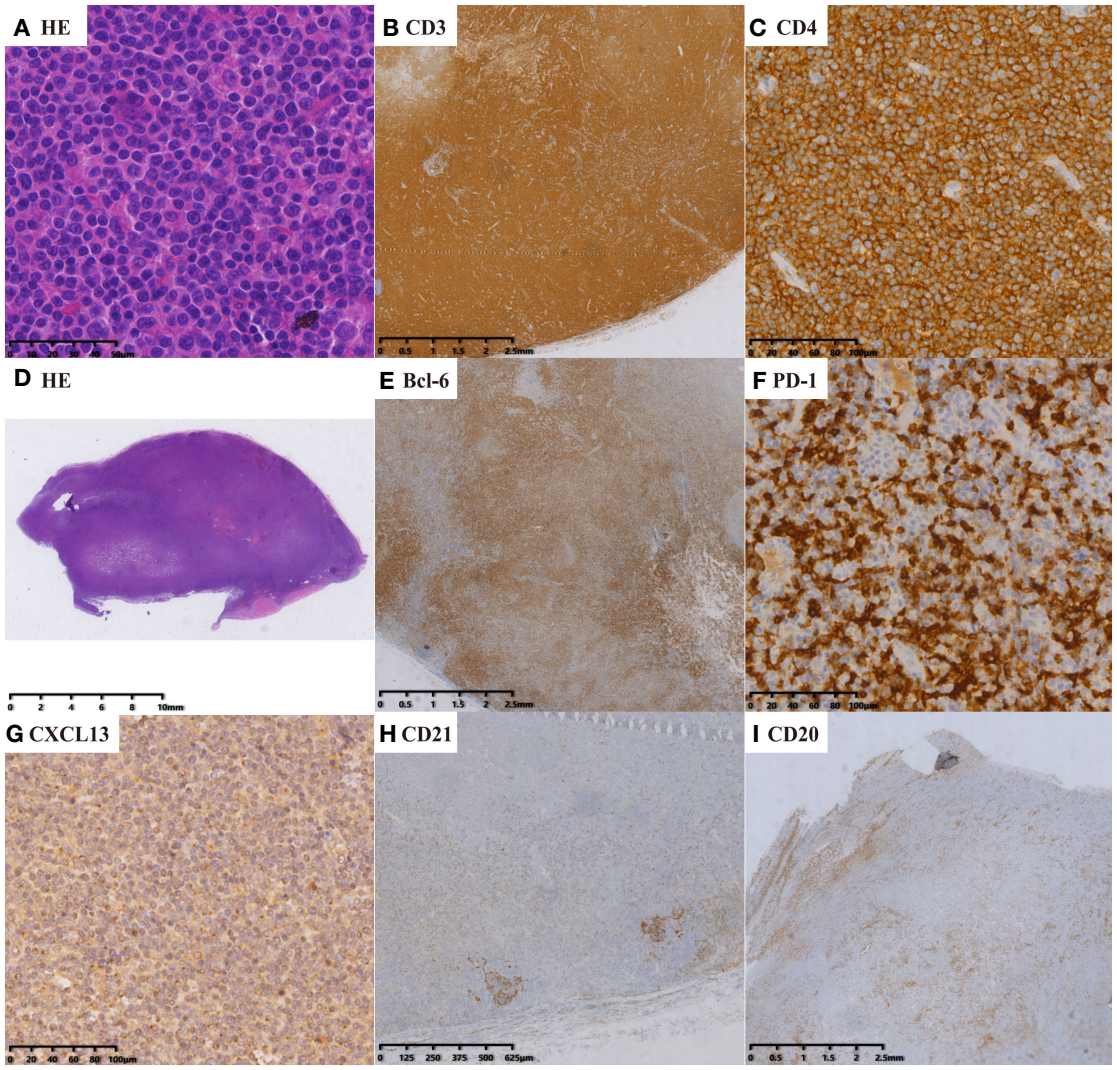
nTFHL-NOS: H&E staining showed that the nodal structure was destroyed, and the tumor cells are diffusely infiltrative (**D** × 4, **A** × 400). IHC staining showed that the tumor cells express CD3 (**B** × 10), CD4 (**C** × 100), BCL-6 (**E** × 10), PD-1 (**F** × 100) and CXCL13 (**G** × 100). A small amount of residual atrophic FDC are positive for CD21 (**H** × 10).

Expert hematopathologists from the pathology department of each participating center reviewed the pathological specimens, including slides stained with hematoxylin-eosin (H&E), immunohistochemistry, and *in situ* hybridization for Epstein–Barr virus encoded RNA (EBER-ISH), at the time of enrollment to confirm the diagnosis. The diagnosis of nTFHL-AI was defined as the destruction of lymph nodal structure accompanied by dendritic hyperplasia of high endothelial venules (HEVs), the proliferation and expansion of follicular dendritic cell (FDC) networks, and a polymorphic inflammatory background containing histiocytes, plasma cells, and eosinophils (**Figure 1**). The diagnosis of nTFHL-F required a follicular growth pattern and FDC meshwork associated with follicles without extrafollicular FDC expansion. The follicles were filled with abnormal T cells expressing TFH markers (**Figure 2**). The diagnosis of nTFHL-NOS was assigned to CD4+ nPTCL with a TFH phenotype that did not meet the criteria for nTFHL-AI and nTFHL-F. This condition usually refers to the absence of dendritic hyperplasia of HEVs, proliferative expansion of the FDC network, and a complex inflammatory background (**Figure 3**). Comprehensive diagnostic criteria were applied, incorporating clinical presentation, morphological features, and immunohistochemical findings. Genetic rearrangement testing was performed when diagnostic uncertainty persisted to facilitate a definitive diagnosis (2, 10–12).

The specimen was fixed in 3.7% neutral formaldehyde, routinely deparaffinized, embedded in paraffin, and cut into 3 μm sections for histological examination using hematoxylin and eosin staining.Immunohistochemical staining was performed using the following antibodies: CD3, CD20, CD5, CD4, CD8, CD10, CD21, BCL-6, CXCL13, PD1, CD30, and Ki-67. The basic information of the antibodies is summarized in the **Supplementary Table S2**. Positive results were determined as follows: PD-1 exhibited cytoplasmic brown-yellow staining, CD10 showed cytoplasmic/membranous brown-yellow staining, bcl-6 exhibited nuclear brown-yellow staining, and CXCL-13 exhibited perinuclear punctate brown-yellow granular staining. The positivity for TFH markers was defined as the expression in at least 10% of tumor cells.

### EBER detection by *in situ* hybridization

EBV status was determined by ISH to detect EBV-encoded RNA 1 and 2 (EBER1/2s) using peroxidase-labeled probes ISH-7001UM (Beijing Zhongshan Golden Bridge Biotechnology, Beijing, China). Tissues from an EBV-positive NK/T cell lymphoma were used as a positive control, and PBS buffer as a negative control in place of primary antibody or hybridization solution. Positive cells (PCs) were defined as the presence of moderate or higher intensity staining (brown/dark brown) in the nucleus. The number of PCs was recorded in high-power fields (HPFs). we defined EBER-positive neoplastic cells per high-power field (HPF) exceeded 50 (> 50/HPF) as EBER(+). Samples with scattered (the number of positive neoplastic cells in the highest density region was less than 50/HPF), inadequate, or equivocal positive neoplastic cells (a cutoff value < 50/HPF) were as EBER (–).

### T-cell and B-cell clonality analysis

Polymerase chain reaction (PCR) amplification of T-cell receptor genes and immunoglobulin gene rearrangements were performed for detecting monoclonality when necessary following the BIOMED-2 protocol as described previously (13). DNA was extracted from the QIAamp DNA Mini Kit (QIAGEN, Hamburg, Germany) following the manufacturer’s protocol. Commercial BIOMED-2 multiplex PCR kits (Righton Gene, Shanghai, China) were used for PCR amplification of TCR and IG gene rearrangements. After separation by capillary electrophoresis, PCR products were subjected to GeneScan analysis on an ABI 9700Genetic Analyzer (Applied Biosystems, CA, USA) and analyzed using the GeneMapper software (Version 4.0; Applied Biosystems, CA, USA).

### Clinical data collection

Clinical data were collected through the use of case report forms. The clinical data included age, sex, B symptoms, Eastern Cooperative Oncology Group (ECOG) performance status, immune inflammatory-related symptoms, Ann Arbor stage, extranodal involvement sites, the International Prognostic Index (IPI), the prognostic index for peripheral T-cell lymphoma unspecified (PIT), treatment methods, treatment response, survival status, and cause of death. The recorded laboratory data included baseline complete blood cell count, albumin level, globulin level, serum lactate dehydrogenase (LDH) level, C-reactive protein (CRP) level, serum immunoglobulin level, Coombs test, and ANA test. Bone marrow involvement was diagnosed through bone marrow biopsy. The involvement of the spleen and other extranodal sites was determined using diagnostic tools such as CT, enhanced CT, and PET-CT. Treatment response was evaluated according to the 2014 Lugano classification criteria (14). The response was categorized as complete remission (CR), partial remission (PR), stable disease (SD), or progressive disease (PD). The objective response rate (ORR) was defined as the proportion of patients who achieved PR and CR.

### Statistical analysis

Quantitative data are expressed as either the mean ± standard deviation (SD) or median with interquartile range (IQR). Intergroup comparisons were conducted using Student’s t test, the Mann−Whitney U test, or the Kruskal−Wallis test, as appropriate. Categorical data are reported as percentages or proportions, and comparisons were made using either Pearson’s chi-square test or Fisher’s exact test. Overall survival (OS) was defined as the time interval from diagnosis to either death from any cause or the last follow-up. The time to disease progression (TTP) was defined as the duration from the initiation of treatment to disease progression. Progression-free survival (PFS) was defined as the period from the commencement of treatment to either disease progression or death from any cause. Survival analysis was conducted utilizing the Kaplan−Meier method, and the log-rank test was used to compare survival rates between the two groups. A *P value* < 0.05 was considered to indicate statistical significance. The statistical analysis was conducted using SPSS software, specifically version 26.0.

## Results

### Patient characteristics

**Table 2** summarizes the baseline characteristics of the entire study population. The nTFHL-NOS patients were younger at disease onset (62.5 vs. 59.0 years, *P=*0.134) than the nTFHL-AI patients were, and they presented less frequently with ECOG score > 1, B symptoms, rash, and serosal effusion. Laboratory and imaging tests revealed a decreased incidence of anemia, decreased albumin levels, elevated globulin levels, elevated LDH levels, elevated CRP levels, increased polyclonal serum immunoglobulin levels, and advanced disease stage. Most patients demonstrated low to intermediate risk according to both the IPI and the PIT score, and these differences were statistically significant. However, there were no statistically significant differences in clinical presentation, laboratory or imaging findings, staging, IPI score, or PIT score between the nTFHL-F cohort and the nTFHL-AI cohort as well as the nTFHL-NOS cohort.

**Table 2:** Characteristics of all patients.

	nTFHL-AI (*n* = 188)	nTFHL-F (*n* = 10)	nTFHL-NOS (*n* = 30)	*P* values		
				AI vs. F	AI vs. NOS	F vs. NOS
Age, years						
Mean	62.5 ± 10.8	62.4 ± 8.3	59 ± 16.5	0.984	0.134	0.537
Older than 60, n% (n/N)	57% (107/188)	60% (6/10)	50% (15/30)	0.999	0.502	0.721
Sex, Male, n% (n/N)	62% (117/188)	40% (4/10)	67% (20/30)	0.191	0.218	0.159
ECOG score > 1, n% (n/N)	42% (78/188)	40% (4/10)	13% (4/30)	0.999	**0.004**	0.089
B-symptoms present, n% (n/N)	48% (90/188)	30% (3/10)	13% (4/30)	0.340	**0.000**	0.338
Rash, n% (n/N)	28% (52/188)	10% (1/10)	10% (3/30)	0.294	**0.042**	0.999
Serous cavity effusion, n% (n/N)	60% (113/188)	30% (3/10)	20% (6/30)	0.096	**0.000**	0.665
Presence of anemia, n% (n/N)	67% (124/188)	70% (7/10)	47% (14/30)	0.999	**0.042**	0.281
Platelet count <100x10^9/L, n% (n/N)	32% (60/188)	40% (4/10)	17% (5/30)	0.730	0.131	0.190
Albumin level < 35 g/L, n% (n/N)	59% (111/188)	40% (4/10)	30% (9/30)	0.326	**0.003**	0.700
Globulin level > 35 g/L, n% (n/N)	33% (62/188)	10% (1/10)	10% (3/30)	0.174	**0.010**	0.999
Elevated LDH level, n% (n/N)	75% (136/182)	88% (7/8)	43% (13/30)	0.682	**0.001**	0.700
CRP level > 2.00 mg/dL, n% (n/N)	86% (130/151)	80% (4/5)	35% (8/23)	0.537	**0.000**	0.133
Positive Coombs test, n% (n/N)	50% (20/40)	67% (2/3)	17% (1/6)	0.999	0.198	0.226
Positive ANA, n% (n/N)	41% (35/85)	33% (1/3)	14% (1/7)	0.999	0.240	0.999
Elevated poly-serum immunoglobulins	41% (35/85)	29% (2/7)	13% (2/15)	0.698	**0.046**	0.565
IgG > 1700 mg/dL, n% (n/N)	53% (45/85)	29% (2/7)	27% (4/15)	0.257	0.077	0.244
IgM > 200 mg/dL, n% (n/N)	53% (45/85)	29% (2/7)	13% (2/15)	0.158	**0.005**	0.565
IgA > 400 mg/dL, n% (n/N)	20% (17/85)	43% (3/7)	7% (1/15)	0.172	0.215	0.077
Stage, III/IV, n% (n/N)	95% (176/186)	90% (9/10)	77% (23/30)	0.447	**0.001**	0.653
No. of extranodal sites > 1, n% (n/N)	34% (62/180)	43% (3/7)	38% (11/29)	0.696	0.715	0.999
Spleen invasion, n% (n/N)	35% (64/182)	0% (0/7)	31% (9/29)	0.097	0.664	0.156
Extranodal involvement, n% (n/N)						
Liver, n/N (%), n% (n/N)	3% (6/182)	0% (0/7)	3% (1/29)	0.999	0.999	0.999
Skin, n/N (%), n% (n/N)	8% (14/182)	29% (2/7)	10% (3/29)	0.110	0.711	0.224
Lung, n/N (%), n% (n/N)	9% (16/182)	0% (0/7)	3% (1/29)	0.999	0.479	0.999
Bone marrow, n% (n/N)	38% (62/162)	78% (7/9)	18% (5/28)	**0.032**	**0.037**	**0.002**
IPI score						
0/1 (low risk), n% (n/N)	10% (17/178)	10% (1/10)	36% (10/28)	0.999	**0.042**	0.278
2 (low-intermediate risk), n% (n/N)	24% (43/178)	20% (2/10)	18% (5/28)			
3 (high-intermediate risk), n% (n/N)	25% (44/178)	20% (2/10)	29% (8/28)			
4/5 (high risk), n% (n/N)	42% (74/178)	50% (5/10)	18% (5/28)			
PIT score						
0 (low risk), n% (n/N)	5% (8/171)	10% (1/10)	30% (9/28)	0.504	**0.001**	0.267
1 (low-intermediate risk), n% (n/N)	26% (44/171)	22% (3/10)	33% (9/28)			
2 (high-intermediate risk), n% (n/N)	33% (56/171)	11% (0/10)	26% (7/28)			
3/4 (high risk), n% (n/N)	37% (63/171)	60% (6/10)	11% (3/28)			

AI, nTFHL-AI; F, nTFHL-F; NOS, nTFHL-NOS; ECOG score, Eastern Cooperative Oncology Group score; LDH, serum lactate dehydrogenase; CRP, C-reactive protein; IPI score, the International Prognostic Index score; PIT score, the prognostic index for peripheral T-cell lymphoma unspecified score; Bold values were statistically significant (P < 0.05).

### Treatment response and survival analysis

**Figure 4:**
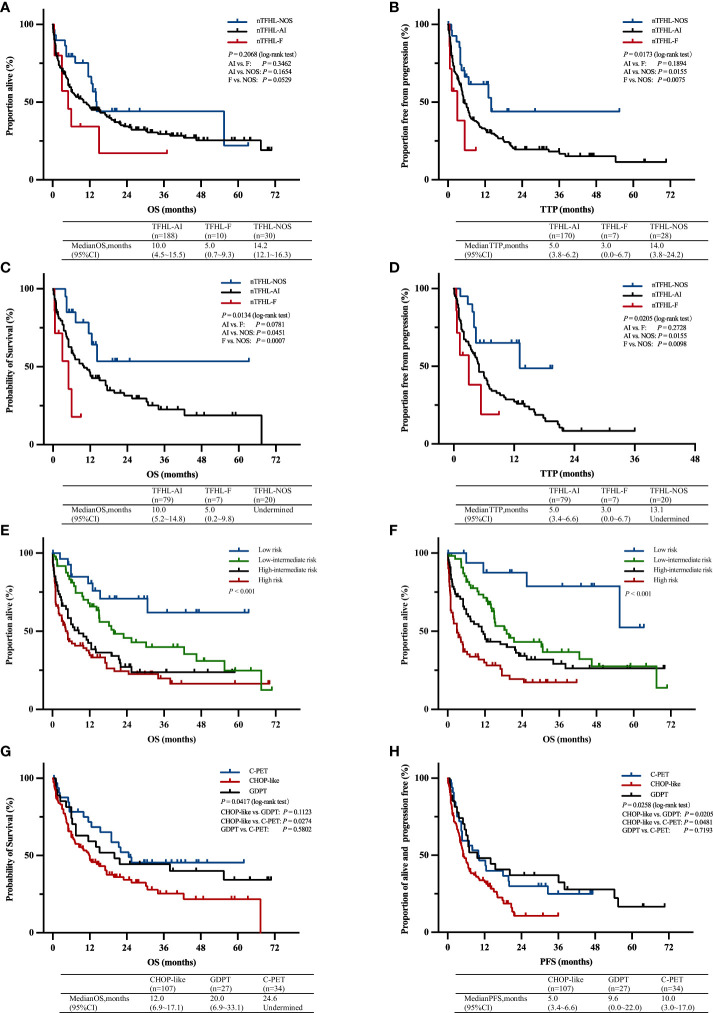
OS **(A)** and TTP **(B)** Kaplan–Meier survival curves of all patients; OS **(C)** and TTP **(D)** Kaplan–Meier survival of all patients with CHOP-like induction therapy; Kaplan–Meier survival curves of OS according to prognostic index for T-cell lymphoma; IPI **(E)**, PIT **(F)**; Kaplan–Meier survival curves of OS **(G)** and PFS **(H)** of all patients with C-PET, GDPT and CHOP-like induction therapy. AI, nTFHL-AI; F, nTFHL-F; NOS, nTFHL-NOS; CHOP, Cyclophosphamide, Doxorubicin, Vincristine, Prednisone; GDPT, Gemcitabine, Cisplatin, Prednisone, Thalidomide; C-PET, Chidamide plus Prednisone, Etoposide, and Thalidomide.

A majority of the patients underwent CHOP-like induction therapy. The ORR for nTFHL-AI, nTFHL-F, and nTFHL-NOS were 41% (25/64), 29% (2/7), and 75% (9/12), respectively (**Table 3**). The GDPT and C-PET regimens demonstrated improvements in the ORR (*P*=0.0141), OS (*P*=0.0417, **Figure 4G**), and PFS (*P*=0.0258, **Figure 4H**) for nTFHLs compared to the CHOP-like regimen. The median OS for the CHOP-like, GDPT, and C-PET regimens were 12 months (95% CI 6.9–17.1), 20.0 months (95% CI 6.9–33.1), and 24.6 months (95% CI undermined), respectively, while the median PFS were 5 months (95% CI 3.4–6.6), 9.6 months (95% CI 0.0–22.0), and 10 months (95% CI 3.0–17.0).

**Table 3:** ORR of all patients’ induction therapy.

	nTFHL-AI (*n* = 188)	nTFHL-F (*n* = 10)	nTFHL-NOS (*n* = 30)	*P* values		
				AI vs. F	AI vs. NOS	F vs. NOS
CHOP-like, n% (n/N)	41% (25/64)	29% (2/7)	75% (9/12)	0.701	**0.022**	0.074
CHOP-like+Novel, n% (n/N)	50% (8/16)	NA	50% (4/8)	0.999	NA	NA
Other						
GDPT, n% (n/N)	62% (13/21)	NA	80% (4/5)	0.621	NA	NA
C-PET, n% (n/N)	72% (20/28)	NA	NA	NA	NA	NA

AI, nTFHL-AI; F, nTFHL-F; NOS, nTFHL-NOS; CHOP, Cyclophosphamide, Doxorubicin, Vincristine, Prednisone; GDPT, Gemcitabine, Cisplatin, Prednisone, Thalidomide; C-PET, Chidamide plus Prednisone, Etoposide, and Thalidomide; Novel, Novel Drugs; Bold values were statistically significant (*P* < 0.05).

With a median follow-up of 36.1 months, the median OS for patients with nTFHL-AI, nTFHL-F, and nTFHL-NOS were 10 months (95% CI 4.5-15.5), 5 months (95% CI 0.7-9.3), and; 14.2 months (95% CI 12.1-16.3), respectively (**Figure 4A**), and the median TTP was 5 months (95% CI 3.8-9.2), 3 months (95% CI 0.0-6.7), and 14 months (95% CI 3.8-24.2). The results of the log-rank test for the nTFHL-AI, nTFHL-F, and nTFHL-NOS subtypes of TTP survival curves showed an overall difference in prognosis (*P*=0.0173, **Figure 4B**). The survival of the nTFHL-NOS subtype was significantly better than that of the nTFHL-AI subtype (*P*=0.0155, **Figure 4B**) and the nTFHL-F subtype (*P*=0.0075, **Figure 4B**). Additionally, in the group of patients receiving induction therapy with CHOP-like regimens, significant differences were observed in OS (*P*=0.0134, **Figure 4C**) and TTP (*P*=0.0205, **Figure 4D**) among the three subtypes. Both the IPI and PIT score retained prognostic value in assessing the outcome of nTFHLs (IPI, *P*<0.001; **Figure 4E**; PIT, *P*<0.001; **Figure 4F**).

### Immunohistochemistry and *in situ* hybridization

Similarly, compared with the nTFHL-AI subgroup, the nTFHL-NOS subgroup displayed relatively lower expression levels of TFH markers, particularly for BCL-6, CD10, and CXCL13. Additionally, the nTFHL-NOS subgroup had a lower median Ki67 index (**Table 4**).

**Table 4:** IHC and EBER-ISH characteristics of all patients.

Pathology finding	nTFHL-AI (n = 188)	nTFHL-F (n = 10)	nTFHL-NOS (n = 30)	*P* values		
				AI VS F	AI VS NOS	F VS NOS
PD-1 positive, n% (n/N)	97.8% (175/179)	100%(9/9)	89.7%(26/29)	0.999	**0.**058	0.999
BCL-6 positive, n% (n/N)	88.4%(152/175)	100%(8/8)	70.4%(19/27)	0.595	**0.027**	0.154
CD10 positive, n% (n/N)	65.4%(121/185)	50.0%(5/10)	33.3%(9/27)	0.329	**0.001**	0.454
CXCL13 positive, n% (n/N)	66.7%(108/162)	70.0%(7/10)	85.7%(24/28)	0.999	**0.047**	0.351
TFH signal number, Median (Range)	3(2;4)	3(2;4)	2(2;4)	0.517	**0.002**	0.196
EBER-ISH Positive, n% (n/N)	64.2%(113/176)	42.9%(3/7)	51.7%(15/29)	0.262	0.218	0.999
Median ki67 index, Median (Range)	60 (10~90)	60(10;75)	45 (20~80)	0.904	0.099	0.584
Ki67 index > 60%, n% (n/N)	27.5%(49/138)	37.5%(3/8)	10.0%(3/30)	0.999	**0.005**	0.094

AI, nTFHL-AI; F, nTFHL-F; NOS, nTFHL-NOS; Bold values were statistically significant (*P* < 0.05).

## Discussion

PTCLs encompasse a heterogeneous group of tumors. Recently, as TFH cell has gained increased recognition, a subset of lymphomas originating from nodal TFH cells has been identified within PTCLs. In contrast to other T-cell subsets, TFH cells undergo multistep differentiation to acquire a unique phenotype characterized by the expression of PD-1, BCL-6, CXCL13, CD10, and ICOS (12). TFH cells located in the B-cell zone of lymph nodes interact with B cells in the GC, promoting B-cell growth, antibody formation, and immunoglobulin class switching. nTFHLs exhibit unique histopathological features and clinical symptoms associated with immune dysregulation. A prime example is nTFHL-AI, which manifests with systemic symptoms, including generalized lymphadenopathy, hepatosplenomegaly, rash, polyserositis, and elevated serum globulin levels. However, the survival and prognostic characteristics of nTFHLs are better than those of PTCL, NOS (15, 16). Since the 4th edition of the WHO Classification of Hematolymphoid Tumors identified three types of nTFHLs, the rarity of the two new subtypes has led to a scarcity of large-scale studies delineating their biological boundaries. The 5th classification continues to designate these types as provisional subtypes, warranting further research. Our study represents the first and largest dataset analysis directly comparing the three subtypes of nTFHLs (10).

In recent years, studies have demonstrated that, compared with nTFHL-AI, nTFHL-NOS is indicative of a relatively indolent subtype (16). Our findings corroborate this statement, as our study revealed that patients with nTFHL-NOS exhibited milder clinical manifestations, fewer laboratory and imaging abnormalities, less advanced-stage disease, and improved treatment response and survival. Furthermore, several case reports have substantiated these findings (17–21). Despite the absence of significant differences in clinical features between nTFHL-AI patients and nTFHL-F patients as well as nTFHL-NOS patients, patients with nTFHL-F exhibited worse survival outcomes, possibly indicating a more unfavorable prognostic subtype. The IPI and PIT scores retain their significance in assessing the prognosis of nTFHLs (22).

Treatment for nTFHLs currently relies on the principles of PTCLs treatment, with CHOP-like regimens being commonly used for induction treatment. However, the response rates and duration of response to CHOP-like regimens are poor, particularly in nTFHL-F patients, for whom only a 29% ORR was observed. Therefore, there is a crucial need to explore new regimens suitable for treating nTFHLs. Advances in molecular biology, epigenetics, and the immunological microenvironment in nTFHLs have led to the study of targeted therapy against nTFHLs’ antibodies, TCR signaling pathways, as well as epigenetic mutations, and immune therapy aimed at regulating the immunological microenvironment (23, 24). Notably, targeted therapy against epigenetic mutations has shown significant benefits, as observed in various clinical studies. Belinostat combined with CHOP or Chidamide combined with CHOP achieved an ORR of 90% in untreated AITL patients. Azacitidine achieved an ORR of 75% and a median PFS of 15 months in R/R AITL patients. In a multicenter phase II study, Azacitidine and Romidepsin achieved an ORR of 80% (12/15) and a CRR of 60% (9/15) in T-FHCL patients. Our center has also conducted multiple clinical trials (25, 26) showing promising results with certain regimens, such as “GDPT” (gemcitabine, cisplatin, prednisone, thalidomide) and “C-PET” (chidamide plus prednisone, etoposide, and thalidomide), which significantly improved disease remission rates and prolonged disease remission duration in nTFHL patients. Furthermore, ongoing research on the new regimen “ Das + AZA + CEP “ (Dasatinib, Azacitidine, Etoposide, Cyclophosphamide, Prednisone) for AITL treatment and the exploration of other new regimens aim to further enhance the therapeutic efficacy of this disease family.

Compared with nTFHL-AI, nTFHL-NOS exhibited lower expression levels of TFH signal, which is consistent with findings from previous studies (15, 16, 27). Additionally, further observation revealed that nTFHL-NOS patients had a lower median Ki67 index, indicating decreased proliferative activity. Recent studies have revealed that the predominant gene mutations in nTFHL-AI include RHOA, TET2, DNMT3A, and IDH2^R172^ mutations, among others, whereas nTFHL-F and nTFHL-NOS exhibit distinct mutation rates. IDH2^R172^, in particular, appears to be exclusive to nTFHL-AI (5, 27–29). Furthermore, studies have demonstrated a correlation between IDH2 and the tumor microenvironment (TME) in patients with nTFHL-AI (7, 30), suggesting a role for epigenetic mutations in the variation of clinicopathological features. Similarly, nTFHL-F is characterized by distinct ITK-SYK fusion genes (31) known to induce malignant PTCL (32). However, due to limitations in the available research data, further investigations of molecular genetics were not conducted. Future research should prioritize the enhancement of molecular and epigenetic investigations of the three subtypes to reveal their underlying pathology and clinical characteristics in greater detail.

Ultimately, notable distinctions exist in terms of clinical manifestations, pathology, and survival outcomes among the three types of nTFHLs. To gain a deeper understanding of the pathological basis and clinical characteristics of the three types of tumors, additional case studies and molecular genetic confirmation are needed.

## Data availability statement

The raw data supporting the conclusions of this article will be made available by the authors, without undue reservation.

## Ethics statement

The studies involving humans were approved by the Ethics Committee of Scientific Research/Medicine Clinical Trial of The First Affiliated Hospital of Zhengzhou University. The studies were conducted in accordance with the local legislation and institutional requirements. Written informed consent for participation was not required from the participants or the participants’ legal guardians/next of kin in accordance with the national legislation and institutional requirements. Written informed consent was obtained from the individual(s) for the publication of any potentially identifiable images or data included in this article.

## Author contributions

SM: Methodology, Formal analysis, Writing – review & editing. SL: Project administration, Software, Writing – original draft. XZu: Writing – original draft, Resources, Investigation, Data curation. WCL: Writing – original draft, Resources, Investigation, Data curation. LW: Writing – original draft, Resources, Investigation, Data curation. ZW: Writing – original draft, Resources, Investigation, Data curation. WPL: Writing – original draft, Resources, Investigation, Data curation. WS: Writing – original draft, Resources, Investigation, Data curation. YW: Writing – original draft, Investigation, Data curation. XZh: Writing – original draft, Investigation, Data curation. MZ: Writing – review & editing, Supervision, Funding acquisition, Conceptualization.

## Supplementary material

Supplementary Table 1 Participating organizations and contact authors

Supplementary Table 2 Details in primary antibodies in immunohistochemical staining
