# Supplementary material for: Clinicopathologic analysis of nodal T-follicular helper cell lymphomas, a multicenter retrospective study from China
Source: Front Immunol. 2024 Mar 27;15:1371534. doi: 10.3389/fimmu.2024.1371534 (PMC11004360; doi:10.3389/fimmu.2024.1371534)
Supplement: Supplementary Table 1 — Participating organizations and contact authors [file Table_1.docx]

| Table S1:Participating organizations and contact authors | | |
| --- | --- | --- |
| **Organization** | **Author** | **E-mail** |
| the First Affiliated Hospital of Zhengzhou University | Mingzhi Zhang | mingzhi_zhang1@163.com |
| Beijing Boren Hospital | Xiaona Zuo | xiaonazuo@163.com |
| Henan Province People’ Hospital | Lifu Wang | WangLF82228@zzu.edu.cn |
| Xijing Hospital, the Fourth Military Medical University | Zhe Wang | zhwang@fmmu.edu.cn |
| West China Hospital of Sichuan University | Wei ping Liu | liuweiping2001@ vip.sina.com |
| Xuzhou Medical University | Wei Sang | xyfylbl515@xzhmu.edu.cn |

| Table S2: Details in primary antibodies in immunohistochemical staining | | | | | |
| --- | --- | --- | --- | --- | --- |
| Primary antibody | Clone | Manufacturer | Specified target | Location | Dilution |
| CD3ε | A0452 | Fuzhou Maixin Biotechnology, fuzhou,China | T cell or Tumor cell | Cytoplasm | 1:100 |
| CD20 | L26 | Fuzhou Maixin Biotechnology, fuzhou,China | B cell | Cell membrane | 1:100 |
| CD4 | UMAB64 | Fuzhou Maixin Biotechnology, fuzhou,China | T helper cell or Tumor cell | Cell membrane | 1:50 |
| CD10 | 56C6 | Beijing Zhongshan Golden Bridge Biotechnology, Beijing, China | TFH cell or Tumor cell | Cell membrane | 1:150 |
| CD21 | EP64 | Fuzhou Maixin Biotechnology, fuzhou,China | Follicular dendritic cell | Cell membrane | 1:100 |
| CD30 | JCM182 | Beijing Zhongshan Golden Bridge Biotechnology, Beijing, China | Immunoblastic cell | Cell membrane | 1:50 |
| BCL6 | ZR280 | Beijing Zhongshan Golden Bridge Biotechnology, Beijing, China | TFH cell or Tumor cell | Cell nuclear | 1:100 |
| CXCL13 | 53610 | Fuzhou Maixin Biotechnology, fuzhou,China | T cell or Tumor cell | Cytoplasm | 1:100 |
| Ki-67 | MIB1 | Fuzhou Maixin Biotechnology, fuzhou,China | Tumor cell | Cell nuclear | 1:150 |
| PD-1 | UMAB199 | Beijing Zhongshan Golden Bridge Biotechnology, Beijing, China | TFH cell or Tumor cell | Cell membrane | 1:50 |
